# X as a Passive Sensor to Identify Opinion Leaders: A Novel Method for Balancing Visibility and Community Engagement

**DOI:** 10.3390/s24020610

**Published:** 2024-01-18

**Authors:** Marco Furini

**Affiliations:** Department of Communication and Economics, University of Modena and Reggio Emilia, 42121 Reggio Emilia, Italy; marco.furini@unimore.it

**Keywords:** opinion leaders, social media communication, community engagement

## Abstract

The identification of opinion leaders is a matter of great significance for companies and authorities, as these individuals are able to shape the opinions and attitudes of entire societies. In this paper, we consider X (formerly Twitter) as a passive sensor to identify opinion leaders. Given the unreliability of the traditional follower count metric due to the presence of fake accounts and farm bots, our approach combines the measures of visibility and community engagement to identify these influential individuals. Through an experimental evaluation involving approximately 4 million tweets, we showed two important findings: (i) relying solely on follower count or post frequency is inadequate for accurately identifying opinion leaders, (ii) opinion leaders are able to build community and gain visibility around specific themes. The results showed the benefits of using X as a passive sensor to identify opinion leaders, as the proposed method offers substantial advantages for those who are involved in social media communication strategies, including political campaigns, brand monitoring, and policymaking.

## 1. Introduction

Social science research has identified the presence of individuals with the capacity to influence others and shape public attitudes, knowledge, and opinions across a spectrum of issues and domains [1,2,3]. Since these individuals possess the power to shape the beliefs of others, they have become highly sought after by companies eager to invest substantial resources in promoting their products or services to audiences of thousands or even millions of followers [4,5].

Studies in the literature have shown that these people operate in various areas, ranging from fashion and consumer decisions to politics and healthcare and suggest possible methods to identify opinion leaders. For instance, the following approaches has been proposed: Positional (this method hinges on the individual’s position rather than their earned respect); Reputational (grounded in nominations from a community, this method identifies individuals esteemed by their peers); Self-designated (individuals nominate themselves as opinion leaders); Sociometrics (this method relies on interviews, querying individuals about their sources of advice and guidance). Each method carries inherent strengths and weaknesses. Consequently, the practical identification of opinion leaders remains a challenge [6].

In this paper, we explore the role of social media as passive sensors in capturing global information without requiring active participation from users [7]. Specifically, we use X (formerly Twitter) as a passive sensor to identify opinion leaders. Our approach is based on the premise that publicly accessible and timestamped tweets provide a rich data source for analyzing human conversations and identifying influential opinion leaders. While the identification of influential accounts on social media is not a novel concept, it is important to note a shift from traditional belief. Previously, the number of followers was deemed a reliable indicator of influence: a larger follower count suggested greater influence [8]. However, recent studies [9,10,11] have highlighted the unreliability of this metric due to artificial inflation caused by fake accounts and bots. A more recent approach involves social network analysis, which maps conversations onto a graph and examines key nodes [12,13]. Yet, this method’s effectiveness is influenced by graph construction, often overlooking platform-specific intricacies like those on X. For instance, graphs based solely on retweets may favor accounts seeking visibility, potentially neglecting those focused on community building. Similarly, graphs reflecting follower/following relationships may disregard interactions, failing to acknowledge that on X, posts can be viewed without following an account [14].

Identifying these influential accounts becomes even more challenging when we consider that they typically fall into two distinct categories: influencers and opinion leaders. Although the literature lacks a precise definition for these categories, we can broadly describe the former as digital entrepreneurs driven by the goal of endorsing brands and selling products on a large scale, while the latter are people who leverage their personality, expertise, and knowledge to shape the opinions or attitudes of others [9,10,15]. Therefore, a key question is on the agenda of many social media managers: how can we identify opinion leaders?

In this paper, our research objective is the identification of opinion leaders. Recognizing these opinion leaders holds significant value in understanding how information circulates on social media, fighting the spread of false and harmful information, and shaping public opinion [11,16]. Indeed, these accounts play a crucial role within social media: they cultivate vibrant communities; they are considered authorities; they are respected and trusted; and they are seen as expert and wise [12,17]. To find these opinion leaders, our hypothesis is that accounts with high engagement levels are more likely to influence public opinion [18]. Although the literature lacks of a universally recognized formula to measure engagement [19,20], an agreed-upon definition suggests that engagement involves spontaneous actions taken by users after reading a post, and the challenge lies in determining which of these actions, or combination thereof, best encapsulates the concept of engagement [18].

In this paper, we propose a novel approach utilizing X as a passive sensor for the identification of opinion leaders. As such, we consider the user’s potential actions such as likes, replies, retweets, and quotes to measure engagements. By assigning appropriate weights to these actions and combining them, our methodology goes beyond traditional approaches that rely solely on follower count. To assess the effectiveness of our method, we performed a comprehensive case study utilizing a dataset consisting of 4 million tweets centered around COVID-19 discussions on X in Italy. The dataset covers the time period from 2020 to 2022. These tweets were obtained using the Academic Twitter API, with a filter applied to include only Italian-written tweets that contained one or more keywords or hashtags relevant to COVID-19.

The outcomes of our study highlight the limitations of relying solely on follower count or post frequency when identifying opinion leaders. In contrast, our proposal strikes a balance by recognizing accounts that not only garner visibility and exposure but also foster community participation. A noteworthy finding is that the opinion leader status is dynamic and time-dependent, aligning with the ever-evolving nature of social media.

Our proposed method represents a step towards more accurate identification of opinion leaders. In particular, this study showed how to use X as a passive sensor. Our approach might be used by researchers and policymakers to develop effective communication strategies and monitor public sentiment regarding brands or products, and it might have broad applications across various domains, including understanding conversation dynamics, political campaigns, brand monitoring, and public opinion analysis.

The remainder of this paper is organized as follows: in Section 2, we present recent literature studies related to the need of metrics to measure social media activities as well as some background related to opinion leaders, influencers, and engagement; Section 3 presents details of our proposal. Section 4 shows the experimental scenario and Section 5 discusses the obtained results. Conclusions are drawn in Section 6.

## 2. Background and Related Work

In this section, we briefly describe studies that focus on the use of social media as passive sensors and the difference between opinion leaders and influencers, we define what is usually referred to by the term engagement, and we briefly review approaches proposed to identify opinion leaders within X.

### 2.1. Social Media Platforms as Passive Sensors

Social media platforms are being used as passive sensors in various fields, including public health, sociology, and political science. This approach allows capturing information about the world without requiring active participation from users. The following studies demonstrate the potential of social media data as passive sensors for monitoring various phenomena. Examples in public health include the analysis of Twitter conversations to monitor the spread of influenza in the United States [21], to track the spread of dengue fever in Brazil [22], and to track health behaviors [23]. Social media platforms as passive sensors have been also used to study social phenomena. Examples include well-being understanding [24], workspace performance [7], understanding of human–environment interactions [25], human pattern identification [26], human mobility [27], air quality monitoring [28], student stress monitoring [29], and misinformation spread during crisis [30].

### 2.2. Opinion Leader vs. Influencer

The term opinion leader commonly refers to individuals who leverage their personality, expertise, and knowledge to influence the opinions and attitudes of others [31,32]. These individuals are recognized as authorities within specific domains and possess the ability to shape the viewpoints of their audience through insightful perspectives and engaging discussions [9,33]. They often act without a direct economic motive [11], and they have an extensive network of relationships across various topics [15].

In contrast, the term influencer generally refers to digital entrepreneurs with a substantial following on social media. Unlike opinion leaders who focus on shaping opinions and attitudes within specific communities, influencers primarily aim to endorse brands and promote products, ideas, or services on a larger scale [10]. Leveraging their online presence, influencers create engaging content tailored to resonate with their audience and utilize their substantial following to effectively market and endorse a wide range of offerings. Their ability to captivate and persuade their followers through strategic content creation and targeted promotional activities makes them a valuable asset for brands seeking to expand their reach and influence on social media.

### 2.3. Social Media Engagement

Measuring social media engagement has become increasingly crucial in contemporary times, with a notable shift in social media campaigns from mere accumulation of views or followers to the encouragement of spontaneous user actions [18]. This shift is primarily driven by the rise of fake followers, emphasizing the importance of evaluating post effectiveness based on active public participation [34]. Generally, higher levels of participation are considered indicative of more effective content [20].

Although the research on this topic has significance in both theoretical and managerial contexts, there is still no consensus on its precise definition [19]. Likely, this ambiguity stems from the fact that engagement draws from diverse fields such as psychology, sociology, and organizational behavior [19,35,36]. Within the realm of social media, engagement has been defined in various ways, including “a positive psychological state of motivation with behavioral manifestations” [37], “the interactive, synchronous communication and collaboration among numerous participants via technology” [38], and “the context-specific occurrence of customer engagement that reflects customers’ individual positive dispositions towards the community or a focal brand” [39]. According to these definitions, engagement encompasses any user action in response to a post, such as liking, commenting, sharing, retweeting, or tagging others.

However, it is crucial to note that not all actions reflect the same level of engagement [40]. Consequently, relying solely on the number of interactions is not right [41]. Instead, engagement is often measured using a weighted combination of numerical features like the number of followers, likes, retweets, mentions, and comments on X [5,42]. Various metrics, including scales, indexes, and action-based metrics, have been developed to measure social media engagement [43]. The existence of multiple metrics is attributed to the unique properties and rules of each social media platform, making a one-size-fits-all metric impossible [44].

### 2.4. X Opinion Leader

Initially, the identification of opinion leaders on X relied on metrics such as follower count or message reach [45]. However, as previously mentioned, this approach is no longer considered valid. Consequently, alternative methods have been explored. One such approach involves the analysis of the language used in tweets, although the brevity of text could potentially lead to misleading results [46]. Another strategy is social network analysis, which is based on the idea that strategically positioned nodes exert influence [12,47,48,49]. While the underlying assumption holds, the results are significantly influenced by graph design. For instance, some studies use retweets or mentions as edges [50,51], while others consider interactions [52], follower strength [53], or simply mentions [14]. A graph solely based on follower/following relationships lacks meaning, since reading messages in X does not require following. On the other hand, a graph centered on mentions does not establish personal relationships, as anyone can mention anyone. Additionally, a graph relying on retweets tend to favor high-follower accounts, potentially neglecting genuine influence.

The diversity in these approaches highlights the complexity of opinion leader identification and the need for nuanced strategies to capture the essence of influence within social media platforms.

## 3. Our Proposal

When developing a method to use X as a passive sensor to identify opinion leaders, a critical starting point involves determining the relative importance of two key account factors: the number of posts published and the level of engagement achieved within a specific time frame. While this decision may seem straightforward, it carries more nuance than it appears. Is an opinion leader someone who posts infrequently but generates high engagement, or is it the opposite? Deliberate thought on these factors is crucial to establishing a reliable metric for identifying opinion leaders.

In the subsequent sections, we will explore diverse approaches to weighting these two factors. Additionally, we will delve into the specifics of measuring engagement within the context of X. Finally, we will provide in-depth details of our proposed metric.

### 3.1. Number of Posts vs. Engagement Score

**Figure 1:**
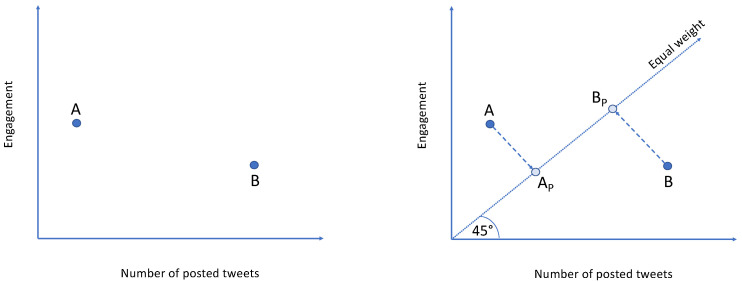
Number of posts versus engagement. Which account, A or B, can truly claim the title of opinion leader? (**Left**). To make a fair assessment, we project them onto the “equal weight” line and gauge their distance from the origin (**right**).

Consider the two accounts illustrated in Figure 1 (left): A exhibits a higher engagement value than B but has published fewer posts. The question arises: which of the two accounts, A or B, can be deemed the opinion leader? If we solely focus on the engagement value, it is unequivocal that account A is the opinion leader, but one might argue that account B is superior, achieving a better balance between posts and engagement.

In general, there is no definitive right or wrong answer, as the weight given to the number of posts and engagement may vary depending on the specific goals and objectives of the social media account or campaign:

- Prioritize number of posts. If the primary goal is to increase visibility and exposure, prioritizing a higher number of content pieces over achieving high engagement may be useful. Examples include politicians or journalists aiming to disseminate propaganda or news rather than forming a community [54].
- Prioritize engagement. If the primary goal is to build a strong community of followers and increase brand loyalty, prioritizing engagement over the number of posts may be appropriate. Examples include fashion brand accounts seeking to create a robust community around their brand.
- Assign equal weight. If the primary goal is to achieve high visibility and build a community, then both factors are equally important and should carry equal weight. Examples include opinion leaders whose goal is to disseminate their opinions while creating a robust community around those opinions.

Among the three potential approaches, we have opted for the latter. This involves assigning equal weight to both the number of posts published and the level of engagement achieved. Our decision is grounded in the belief that an opinion leader strives for both visibility and community engagement [12,20]. Consequently, instead of solely focusing on points A and B (as depicted in Figure 1 (left)), we consider their projections on the “equal weight” line, positioned at a 45-degree angle to the X-axis (as illustrated in Figure 1 (right)). The farther the distance from the XY origin, the more favorable the performance.

### 3.2. X Engagement Score

Up to this point, our focus has been on engagement without elucidating the methodology for its measurement. Needless to say, it is imperative to define the engagement score (Y-axis), as engagement encompasses spontaneous reactions triggered by an event. On the X platform, a post triggers reactions such as like (indicating appreciation for a tweet), reply (responding to another user’s tweet), retweet (circulating content within the user’s follower network), and quote (embedding a tweet within a personal message).

Diverse methods can be employed to amalgamate these actions, ranging from straightforward tallies to intricate formulas that assign varying weights to different interaction types. Here, we introduce the Tweet Engagement Score (TES), a metric designed to compute the engagement score of an individual tweet. The TES not only considers the count of likes but also encompasses other interaction types such as retweets, replies, and quotes. By integrating multiple interaction types and assigning distinct weights to each, the TES provides a more holistic measure of a tweet’s engagement in comparison to metrics that solely focus on one interaction type [14].

It is worth noting that establishing the levels of importance, and consequently the weights, of possible interactions is a process that cannot be trivialized with simple assumptions. It must be approached using real data, which are only available from social media platforms. Such a study goes beyond the scope of this work. Therefore, in this present study, we use weights similar to the ones employed by Facebook [55]:

(1) $$TES\left(K,i\right)=l_{i}+5\times rt_{i}+15\times sr_{i}+20lr_{i}+25\times sq_{i}+30\times lq_{i}$$

where *K* is the account of who posted tweet *i*, and $l_{i},rt_{i},sr_{i},lr_{i},sq_{i}$, and $lq_{i}$ are the number of likes, retweets, short replies, long replies, short quotes, and long quotes that tweet *i* received.

The Tweet Engagement Score can be used to compute the engagement score of a X account in a specific period (Account X Engagement Score) as follows:

(2) $$AXES\left(T,K\right)=\sum_{i=1}^{N}TES(K,i)$$

where *T* is the considered time period, *K* is the account, *N* is the number of posted tweets, and TES(K,i) is the Tweet Engagement Score achieved by tweet *i*.

### 3.3. Opinion Leader Score

**Figure 2:**
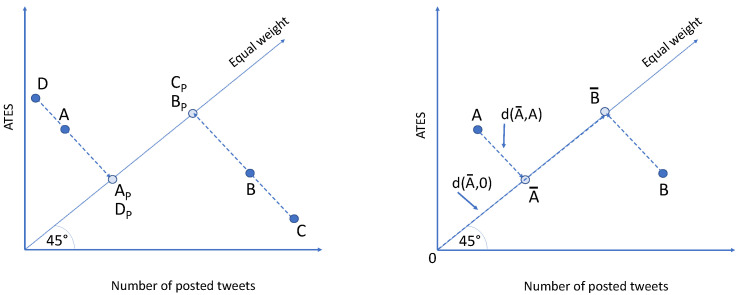
A mere projection is inadequate for grasping the uniqueness of an account (**on the left**). It is essential to take into account the distance of the projection point both from the original point and from the XY origin (**on the right**).

Projecting accounts onto the “equal weight” line requires an improvement. In Figure 2 (left), two additional points, C and D, display different behaviors from A and B, yet their projections are identical. Therefore, it is crucial to factor in the distance of the point from its projection. The closer the point is to its projection, the better its performance. For instance, comparing points C and D, despite sharing the same projection point as A and B, C and D are farther from the desired behavior. Hence, a meticulous consideration of both the projection and its distance is essential for establishing a reliable metric in identifying opinion leaders.

We propose a novel metric called OLS (Opinion Leader Score) of an account *K* in a time period *T* as:

(3) $$OLS\left(T,K\right)=distance\left(0,\bar{K}\right)-distance\left(K,\bar{K}\right)$$

where distance(∗,∗) is the distance between two points, and $\bar{K}$ denotes the projections points of *K* over the “equal weight” line. Figure 2 (right) shows the graphical explanation of Equation (3).

**Figure 3:**
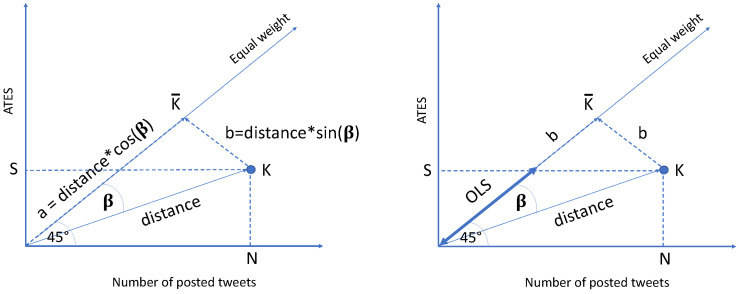
Graphical illustration of the OLS metric involves characterizing an account, denoted as *K*, by considering the number of posted tweets (N) and the engagement score (S). The projection of this account on the ideal line is represented as $\bar{K}$. OLS is then determined as the difference between *a* and *b* in this context.

Mathematically, Equation (3) can be rewritten as:

(4) $$\begin{array}{c} OLS\left(T,K\right)=\left(cos\left(\beta \right)-sin\left(\beta \right)\right)\times \sqrt{N_{K}^{2}+AXES{\left(T,K\right)}^{2}} \end{array}$$

where $N_{K}$ is the number of posted tweets, and AXES(T,K) is the engagement achieved by account *K* in the period *T*. Figure 3 gives a graphical explanation of Equation (3).

The OLS effectively identifies opinion leader accounts by assigning equal weight to both the number of posts and the achieved engagement. This approach offers a comprehensive and nuanced evaluation of an account’s influence as an opinion leader. Through a comparative analysis of OLS values across various accounts, it becomes feasible to establish a ranking of opinion leaders.

## 4. Experimental Analysis

In this section, we apply our proposed method to five datasets encompassing COVID-19 conversations on X in Italy, spanning the years 2020, 2021, and 2022. The objective of this empirical evaluation is to ascertain the efficacy of the proposed method, particularly in comparison to metrics relying on follower counts or interaction frequency. In the following, we delineate the characteristics of the used data, propose a possible classification of accounts into well-known categories, compute the engagement score for all the dataset accounts, and analyze potential correlations with both the followers counts and volume of published posts. Then, we compute the opinion leader score to identify accounts able to influence public opinions. We also analyze the ability to foster community engagement, along with an exploration of their categorized domains of influence.

### 4.1. Dataset

All five datasets have been obtained through the Academic Twitter API, filtering Italian-written tweets containing one or more words/hashtags related to COVID-19. To clarify, tweets must contain terms such as #covid19, #coronavirus, #vaccine, #vaccination, #vax, #novax, #greenpass, #terzadose, #mrna, and #sarscov2. As shown in Table 1, the five datasets collectively provide us with approximately 4 million tweets:

- Arrival (January–May 2020): This period marked the initial significant disruption to Italian daily life due to COVID-19, with the first case officially detected in northern Italy in February. The lockdown, commencing in March, persisted until the end of May.
- Denial (June–December 2020): Skepticism about the virus emerged, with some questioning its existence and claiming the pandemic was a staged event organized by governments.
- Vaccine (January–June 2021): The vaccination campaign in Italy began during this period. Simultaneously, the “novax movement” expressed concerns about the vaccine, including side effects like 5G implantation and alleged death control.
- Greenpass (July–December 2021): The introduction of the “green pass” occurred during this period, serving as a health certificate for those who received two doses of the COVID-19 vaccine. It facilitated a return to everyday life but also sparked protests.
- Post-COVID (January–December 2022): Italy returned to everyday life without restrictions during this phase, signifying the end of the pandemic’s most severe phase.

We selected the COVID-themed dataset because the pandemic has led to widespread social media use across diverse demographics and interests. This includes individuals of all ages, from the young to the elderly, and people from various professional backgrounds, including academics, professionals, humanists, scientists, and individuals with different educational backgrounds. Moreover, spanning three years, the dataset captures the evolving thematic focus over time, encompassing a wide range of interests, from medical information-seeking to social issues, personal freedom, and, post-COVID, a mix of general topics like travel and illnesses.

### 4.2. Account Category

For a deeper understanding of opinion leader accounts, we have defined some categories such as: “Health Community” (accounts affiliated with health authorities, doctors, scientists, and health personnel), “Politics” (accounts of political figures, government or public institutions), “Information” (accounts of newspapers, TV stations, magazines, and blogs), “Very Important People” (accounts of well-known public figures such as actors, sportsmen, and businessmen), and “Ordinary People” (accounts of individuals not fitting into any of the aforementioned categories and not widely recognized).

By mapping the identified opinion leaders into the categories, we might be able to understand if there are categories that are more influential than others.

### 4.3. X Engagement Score

The initial phase in pinpointing opinion leaders involves the computation of the engagement score for each account actively participating in the conversations. This entails determining the AXES value (Equation (2)) for each individual account and the TES value for every tweet within the dataset.

Following the computation of the AXES value for each individual account, the subsequent step is the calculation of the OLS value. Nonetheless, before delving into this calculation, it is prudent to explore the potential existence of a correlation among the number of followers, the count of published posts, and AXES. This analysis aims to offer insights into the factors contributing to an account’s influence as an opinion leader. Understanding these correlations is essential for a comprehensive grasp of the dynamics influencing an account’s status as an opinion leader.

### 4.4. Correlation between Followers and Engagement

To explore potential relationships between the number of followers or published tweets and the AXES engagement score, we conducted a correlation analysis. The findings are presented in Table 2. The results indicate an absence of correlation between the number of followers and the AXES engagement score: in the denial dataset, the correlation between followers and AXES was 0.07, while in the vaccination dataset, it was 0.06; for the greenpass dataset, the correlation was 0.04, and in the post-COVID dataset, it was 0.05. These consistent results affirm that a high number of followers does not guarantee a high engagement score, debunking the notion that an opinion leader can be solely defined by follower count.

A moderate correlation is observed between AXES and the number of posts: 0.18 (arrival), 0.25 (denial), 0.26 (vaccination), 0.32 (greenpass), and 0.22 (post-COVID). This implies that writing numerous posts might help but does not necessarily lead to a high AXES engagement score.

### 4.5. Opinion Leader Score

After calculating the AXES value for each individual account, the subsequent step involves determining its OLS value. Given that this metric entails measuring the distance between two points (see Figure 2), each defined by the pair of the number of published posts and AXES, it is crucial to address issues arising from differences in magnitudes between the two parameters. To overcome this challenge, we opted for data normalization using the Z-score method. This approach ensures that the data have a standard deviation of one and a mean close to zero, facilitating more accurate comparisons and analysis.

Table 3 showcases the top 15 accounts during the “arrival” dataset based on the OLS metric. Notably, the number of followers did not exert influence on the OLS value. For instance, an account with only 5000 followers outperformed an account with over one million followers. The ranking revealed that 10 out of the top 15 accounts were information-based, while two accounts belonged to ordinary individuals and two were health-related accounts. One account was categorized as removed and unknown, but it is plausible that it was an ordinary account posting controversial content. These results underscore the effectiveness of the OLS metric as a potent tool for pinpointing the most influential accounts during the arrival period, regardless of their follower count or the number of posts that they have published.

Table 4 shows the top 15 accounts during the “arrival” dataset ranked solely on the number of interactions. The ranking shows that eight accounts were information-based, two belonged to ordinary individuals, two were health-related accounts, and three were accounts of politicians.

While comparing Table 3 and Table 4 may initially seem challenging, a closer examination reveals the presence of three political accounts in the interaction-based ranking. These accounts provide evidence that relying solely on the number of interactions is insufficient for identifying opinion leaders. Indeed, politician accounts aim to enhance visibility by updating their followers on political initiatives or expressing opinions on specific topics, rather than focusing on community building [56]. Moreover, considering their substantial number of followers, it is more appropriate to classify them as influencers rather than opinion leaders.

The comparison shows that relying solely on the number of interactions can distort perceptions, particularly for accounts belonging to politicians who often accumulate a large number of followers expressing support without actively engaging with the content. By utilizing the OLS approach, we gain a more nuanced understanding of the accounts that hold the most influence in shaping conversations and driving engagement on social media.

In the analysis of other datasets (not presented here for space reasons), a similar pattern emerged, with the ranking based on the number of interactions consistently including political accounts, while the OLS-based ranking excluded them.

### 4.6. Opinion Leader Score Level

**Figure 4:**
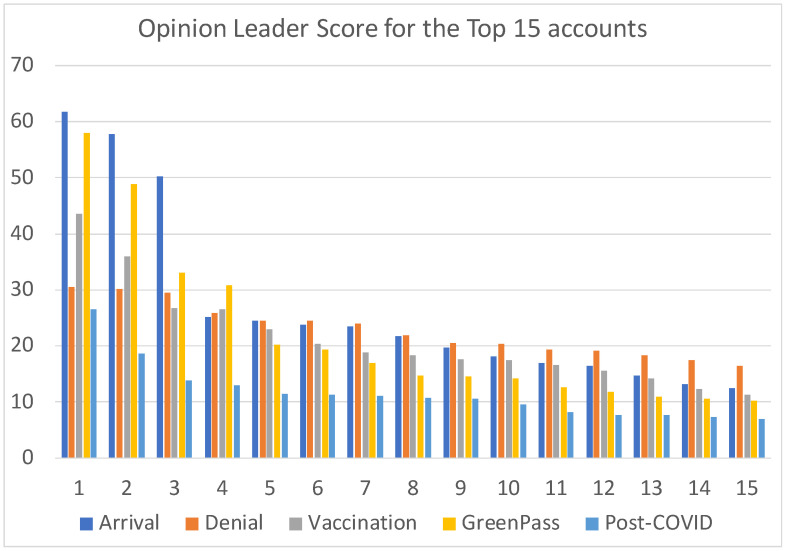
OLS value reached by the top 15 opinion leaders in each of the different dataset analyzed.

Not all opinion leaders are effective in the same way, as some are able to capture more attention than others. The OLS serves as a valuable metric to quantify this. Figure 4 offers a visual comparison of OLS values among the top 15 opinion leaders in each analyzed dataset. In the arrival dataset, OLS values stood out, signaling that these accounts excelled in engaging users on the discussed topics. However, a shift occurred in the post-COVID dataset, where OLS notably decreased. This suggests that opinion leaders were less effective in community-building around the theme.

For a more comprehensive comparison of opinion leaders’ performance across datasets, Table 5 provides statistical insights into OLS. The difference between the arrival and post-COVID is striking (27 vs. 12), emphasizing that opinion leaders were twice as effective in engaging people. Additionally, examining the denial and greenpass datasets reveals intriguing patterns: despite comparable average OLS (23 vs. 22), the highest OLS values diverge significantly (30 vs. 58); this indicates that during the green pass period, some accounts successfully fostered communities, while a notable portion of the top 15 opinion leaders fell short.

### 4.7. Opinion Leader Categories

**Figure 5:**
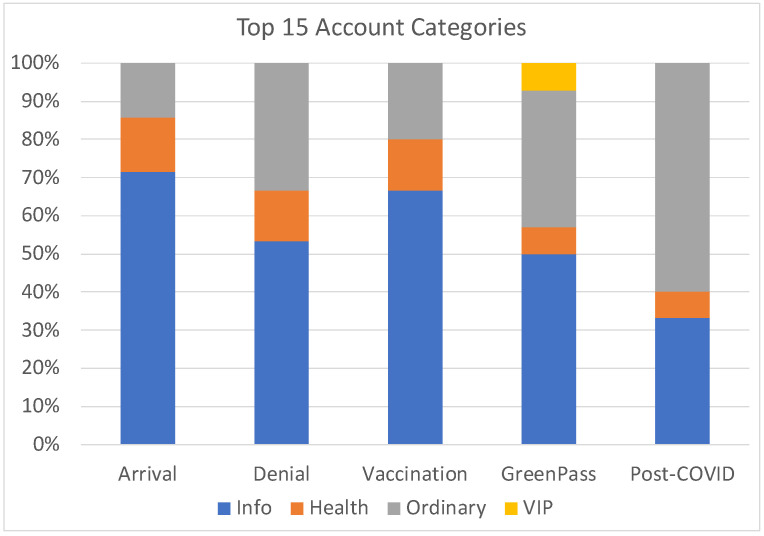
Categories of the top 15 opinion leaders.

Who are the accounts that emerged as opinion leaders? The answer to this question aids in comprehending the dynamics that characterized conversations in distinct periods and discerning dominant categories. Figure 5 shows the categories of the leading 15 opinion leaders in the analyzed datasets. A noteworthy observation is the shifting prominence of the “Ordinary People” category, which exhibited limited presence within the arrival and vaccination datasets but gained significance in the denial, greenpass, and post-COVID datasets. Despite often having a modest following, these accounts demonstrated a capacity to foster engagement and build community around their posts.

Another intriguing finding is the limited presence of accounts in the Health category. Only two accounts succeeded in engaging people in conversations: the personal account of a medical doctor and the official account of a health authority in an Italian region. These accounts were effective in engaging users by providing precise and scientific information about the progression of the epidemic. Despite the remarkable communication efforts of these two health-related accounts, the results underscore the challenge faced by health authorities in effectively engaging users. It is crucial to note that this lack of engagement may contribute to a decrease in trust in science. Indeed, without effective communication from health institutions, there is a risk of misinformation spreading faster and more widely than scientific evidence, posing serious risks to public health.

## 5. Discussion

Our proposed method has both theoretical and practical implications, as discussed in the following.

### 5.1. Theoretical Implications

Our proposal holds theoretical implications, as it underscores that social media platforms like X might be used as passive sensors to identify opinion leaders. Furthermore, our proposal shows that identifying opinion leaders cannot solely rely on the number of followers or the quantity of posts published, aligning with findings from prior researches [10,11]. Moreover, depending solely on the number of interactions may yield interpretations divorced from reality, as it can be influenced by users aiming to boost visibility and mediated exposure (e.g., the presence of politicians when the ranking was based on the number of interactions) [56]. In contrast, our proposed approach is able to catch the peculiarity of an account that tries to build a community around a specific theme. This is further confirmed by the presence of several accounts of ordinary people.

Another noteworthy theoretical contribution emerges from our case study analysis, revealing the limited impact of health-related accounts on conversations due to their struggle to engage users. This finding aligns with prior studies highlighting how healthcare institutions often misapply social media platforms, primarily using them as channels for disseminating news and press releases rather than fostering meaningful conversations and interactions around a topic [38,57,58].

Furthermore, people appear to engage in conversations on a specific topic without necessarily focusing on the author of the message, aligning with insights from prior research [3,59]. Indeed, our study shows that only a handful of accounts maintained their status as opinion leaders across different periods: being an opinion leader in one conversation does not guarantee the same status in subsequent discussions.

### 5.2. Practical Implications

Using X as a passive sensor to identify opinion leaders brings benefits to a diverse spectrum of individuals and entities, encompassing researchers, marketers, journalists, and public authorities [16]. Researchers can leverage this information to delve into the dynamics of opinion formation and information dissemination on X. Scrutinizing the conduct of opinion leaders offers valuable insights into the determinants of public opinion and the efficacy of varied communication strategies [16].

Marketers, armed with knowledge about opinion leaders, can enhance their credibility and optimize strategies for promoting products or services. Journalists, on the other hand, can pinpoint sources for their stories and trace the trajectory of news dissemination through social media channels. Public authorities stand to gain by engaging with opinion leaders, utilizing such interactions to refine their communication strategies, shape public discourse, and advocate for their policies. For instance, our case study showed that health-related accounts were unable to build community around their posts. If OLS were available to them, they could have discerned the ineffectiveness of their communication efforts and identified more influential accounts for conveying their crucial messages. This underscores the practical utility of our proposal in guiding accounts towards more effective communication within the X platform.

### 5.3. Future Research Directions

The OLS metric uses an engagement measure that takes into account both the level of engagement and the number of posts. While we relied on established weights from existing literature [55], it is important to acknowledge that determining these weights is a nuanced task that lacks a one-size-fits-all solution. Weight assignments may vary depending on the type of account or content being analyzed. For instance, news accounts might prioritize retweets, which indicate broader content dissemination, while personal accounts might emphasize replies, signaling more direct engagement with followers.

The tuning of weights is based on considerations aligned with the specific analysis needs, contextual variations, and metric objectives. In this study, our goal was to identify opinion leaders, so we used weights [55] that aimed to capture both visibility/exposure and community building. Future studies could explore adapting these weights for different situations. For instance, when identifying opinion leaders who foster a brand’s community, assigning greater weight to parameters like replies could be more appropriate, while for those emphasizing brand visibility, prioritizing retweets might be more suitable.

Regarding dataset specificity, future investigations could extend our proposal to niche datasets, delving into specific topics such as cryptocurrencies, football, or distinct diseases.

## 6. Conclusions

This study showed how to use X as a passive sensor to identify opinion leaders. We designed a method to analyze X conversations, which effectively combines visibility and community engagement. The empirical results highlight the inadequacy of relying solely on metrics like follower counts or posting frequency to identify opinion leaders, emphasizing the need for a customized metric. Notably, our proposed metric showed superior efficacy in identifying opinion leader accounts compared to approaches solely based on interaction counts. In summary, this study showed that X might be used as a passive sensor to identify opinion leaders. Our proposal represents an initial step in the quest to precisely identify opinion leaders, a pivotal process with practical implications for diverse social participants, including researchers, marketers, journalists, and public authorities.

## Figures and Tables

**Table 1:** Numerical characteristics of the different datasets.

Dataset Name	# of Tweets	# of Unique Accounts	Period
Arrival	1,447,486	143,626	January–May 2020
Denial	696,966	70,619	June–December 2020
Vaccination	526,047	62,810	January–June 2021
Greenpass	762,733	66,686	July–December 2021
Post-COVID	511,268	41,834	January–December 2022

**Table 2:** AXES Correlation with number of followers and number of posts.

Dataset	Correlation	Correlation
Name	AXES-Followers	AXES-Posts
Arrival	0.12	0.18
Denial	0.07	0.25
Vaccination	0.06	0.26
Greenpass	0.04	0.32
Post-COVID	0.05	0.22

**Table 3:** Arrival Dataset: top 15 accounts according to OLS. The word between brackets reveals the account category. Usernames are partially obscured with (***) for privacy reasons.

Username	OLS	Followers	n_interactions	n_post
Adn *** (Info)	61.7	534k	188k	8268
Age *** (Info)	57.7	1433k	558k	5255
ult *** (Info)	50.2	111k	309k	4576
TgL *** (Info)	25.2	693k	184k	2301
Lib *** (Info)	24.4	296k	130k	2231
Med *** (Info)	23.8	1181k	141k	2180
Sal *** (Health)	23.4	15k	59k	2311
rtl *** (Info)	21.8	868k	162k	1996
duk *** (Ordinary)	19.6	12k	45k	1792
Lan *** (Unknown)	18.1	5k	54k	2922
Rai *** (Info)	16.9	1119k	81k	1545
fan *** (Info)	16.4	354k	69k	1505
TGL *** (Info)	14.6	534k	53k	2333
val *** (Ordinary)	13.2	37k	85k	1208
Car *** (Health)	12.5	52k	262k	1150

**Table 4:** Arrival period: top 15 accounts according to the number of interactions received by the account. The word between brackets reveals the account category. Usernames are partially obscured with (***) for privacy reasons.

Username	n_interactions	Followers	n_post
Rad *** (Ordinary)	674,649	44,906	577
Age *** (Info)	558,960	1,433,983	5255
Gio *** (Politics)	370,333	1,127,142	123
ult *** (Info)	309,475	111,967	4576
Min *** (Health)	265,366	273,556	388
Car *** (Health)	262,356	52,484	1150
fra *** (Info)	256,296	26,859	987
Amb *** (Politics)	247,223	36,468	249
Giu *** (Politics)	245,108	1,037,863	40
pie *** (Info)	228,183	27,796	275
gab *** (Ordinary)	210,874	1446	20
Adn *** (Info)	188,588	534,191	8268
TgL *** (Info)	184,542	693,411	2301
sta *** (Info)	170,993	1,067,289	75
you *** (Info)	166,621	90,056	750

**Table 5:** OLS statistical data for the top 15 accounts of the different analyzed datasets.

Dataset	Mean	Median	ST.DEV	Highest	Lowest
Arrival	27	22	16.13	61.7	12.5
Denial	23	22	4.63	30.4	16.4
Vaccination	21	18	8.78	44.5	11.8
Greenpass	22	15	14.67	57.9	10.3
Post-COVID	12	11	5.10	26.4	7.03

## Data Availability

The data that support the findings of this study have been collected through Academic Twitter API.

## References

[1] Katz E., Lazarsfeld P.F. (1956). Personal Influence: The Part Played by People in the Flow of Mass Communications.

[2] Weimann G. (1994). Influentials, The: People Who Influence People.

[3] White R.W., Hassan A. (2014). Content Bias in Online Health Search. ACM Trans. Web.

[4] Ranpariya V., Chu B., Fathy R., Lipoff J.B. (2020). Instagram influencer definitions and the need for dermatologist engagement on social media. J. Am. Acad. Dermatol..

[5] Montangero M., Furini M. TRank: Ranking Twitter users according to specific topics. Proceedings of the 2015 12th Annual IEEE Consumer Communications and Networking Conference (CCNC).

[6] Weimann G., Tustin D.H., van Vuuren D., Joubert J.P.R. (2007). Looking for Opinion Leaders: Traditional vs. Modern Measures in Traditional Societies. Int. J. Public Opin. Res..

[7] Saha K., Bayraktaroglu A.E., Campbell A.T., Chawla N.V., De Choudhury M., D’Mello S.K., Dey A.K., Gao G., Gregg J.M., Jagannath K. Social media as a passive sensor in longitudinal studies of human behavior and wellbeing. Proceedings of the Extended Abstracts of the 2019 CHI Conference on Human Factors in Computing Systems.

[8] Ismail K. (2020). Social Media Influencers: Mega, Macro, Micro or Nano. CMSWire.

[9] Li Y., Ma S., Zhang Y., Huang R. (2013). An improved mix framework for opinion leader identification in online learning communities. Knowl. Based Syst..

[10] Arrami S., Oueslati W., Akaichi J. Detection of Opinion Leaders in Social Networks: A Survey. Proceedings of the International Conference on Intelligent Interactive Multimedia Systems and Services.

[11] Hosseini Bamakan S.M., Nurgaliev I., Qu Q. (2018). Opinion Leader Detection: A Methodological Review. Expert Syst. Appl..

[12] Alexandre I., Jai-sung Yoo J., Murthy D. (2022). Make tweets great again: Who are opinion leaders, and what did they tweet about Donald Trump?. Soc. Sci. Comput. Rev..

[13] Jain L., Katarya R., Sachdeva S. (2023). Opinion Leaders for Information Diffusion Using Graph Neural Network in Online Social Networks. ACM Trans. Web.

[14] Boatwright B.C. (2022). Exploring online opinion leadership in the network paradigm: An analysis of influential users on Twitter shaping conversations around anthem protests by prominent athletes. Public Relat. Rev..

[15] Solomon M. (2007). Consumer Behavior: Buying Having and Being.

[16] Dellmuth L., Shyrokykh K. (2023). Climate change on Twitter: Implications for climate governance research. Wiley Interdisciplinary Reviews: Climate Change.

[17] Liu J., Zhang Z., Qi J., Wu H., Chen M. (2019). Understanding the Impact of Opinion Leaders’ Characteristics on Online Group Knowledge-Sharing Engagement from In-Group and Out-Group Perspectives: Evidence from a Chinese Online Knowledge-Sharing Community. Sustainability.

[18] Xu Q., Yu N., Song Y. (2018). User Engagement in Public Discourse on Genetically Modified Organisms: The Role of Opinion Leaders on Social Media. Sci. Commun..

[19] Trunfio M., Rossi S. (2021). Conceptualising and measuring social media engagement: A systematic literature review. Ital. J. Mark..

[20] Khan M.L. (2017). Social media engagement: What motivates user participation and consumption on YouTube?. Comput. Hum. Behav..

[21] Signorini A., Segre A.M., Polgreen P.M. (2011). The use of Twitter to track levels of disease activity and public concern in the US during the influenza A H1N1 pandemic. PLoS ONE.

[22] Althouse B.M., Ng Y.Y., Cummings D.A. (2011). Prediction of dengue incidence using search query surveillance. PLoS Neglected Trop. Dis..

[23] Saha K., Chan L., De Barbaro K., Abowd G.D., De Choudhury M. (2017). Inferring mood instability on social media by leveraging ecological momentary assessments. Proceedings of the ACM on Interactive, Mobile, Wearable and Ubiquitous Technologies.

[24] Verduyn P., Lee D.S., Park J., Shablack H., Orvell A., Bayer J., Ybarra O., Jonides J., Kross E. (2015). Passive Facebook usage undermines affective well-being: Experimental and longitudinal evidence. J. Exp. Psychol. Gen..

[25] Ghermandi A., Sinclair M. (2019). Passive crowdsourcing of social media in environmental research: A systematic map. Glob. Environ. Chang..

[26] Timokhin S., Sadrani M., Antoniou C. (2020). Predicting venue popularity using crowd-sourced and passive sensor data. Smart Cities.

[27] Furini M., Montangero M. (2023). Twitter as Passive Sensor to Understand How COVID-19 Pandemic Affected Human Mobility. Proceedings of the 2023 IEEE 20th Consumer Communications & Networking Conference (CCNC).

[28] Wang S., Paul M.J., Dredze M. (2015). Social media as a sensor of air quality and public response in China. J. Med. Internet Res..

[29] Saha K., De Choudhury M. Modeling stress with social media around incidents of gun violence on college campuses. Proceedings of the ACM on Human-Computer Interaction.

[30] Lee J., Britt B.C., Kanthawala S. (2023). Taking the lead in misinformation-related conversations in social media networks during a mass shooting crisis. Internet Res..

[31] King C.W., Summers J.O. (1970). Overlap of Opinion Leadership across Consumer Product Categories. J. Mark. Res..

[32] Grimes A.J., Berger P.K. (1970). Cosmopolitan-Local: Evaluation of the Construct. Adm. Sci. Q..

[33] Doehne M., Herfeld C. (2023). How academic opinion leaders shape scientific ideas: An acknowledgment analysis. Scientometrics.

[34] Hollebeek L.D., Glynn M.S., Brodie R.J. (2014). Consumer Brand Engagement in Social Media: Conceptualization, Scale Development and Validation. J. Interact. Mark..

[35] Shawky S., Kubacki K., Dietrich T., Weaven S. (2019). Using social media to create engagement: A social marketing review. J. Soc. Mark..

[36] Santos Z.R., Cheung C.M., Coelho P.S., Rita P. (2022). Consumer engagement in social media brand communities: A literature review. Int. J. Inf. Manag..

[37] Megha S. (2016). A brief review of employee engagement: Definition, antecedents and approaches. Clear Int. J. Res. Commer. Manag..

[38] Heldman A.B., Schindelar J., Weaver J.B. (2013). Social media engagement and public health communication: Implications for public health organizations being truly social. Public Health Rev..

[39] Dolan R., Conduit J., Fahy J., Goodman S. (2017). Social media: Communication strategies, engagement and future research directions. Int. J. Wine Bus. Res..

[40] Schivinski B., Christodoulides G., Dabrowski D. (2016). Measuring consumers’ engagement with brand-related social-media content: Development and validation of a scale that identifies levels of social-media engagement with brands. J. Advert. Res..

[41] Tiago M.T.P.M.B., Veríssimo J.M.C. (2014). Digital marketing and social media: Why bother?. Bus. Horiz..

[42] Leeflang P.S., Verhoef P.C., Dahlström P., Freundt T. (2014). Challenges and solutions for marketing in a digital era. Eur. Manag. J..

[43] Trunfio M., Della Lucia M. (2019). Engaging destination stakeholders in the digital era: The best practice of Italian regional DMOs. J. Hosp. Tour. Res..

[44] Dolan R., Conduit J., Fahy J., Goodman S. (2016). Social media engagement behaviour: A uses and gratifications perspective. J. Strateg. Mark..

[45] Rattanaritnont G., Toyoda M., Kitsuregawa M. (2012). Characterizing topic-specific hashtag cascade in twitter based on distributions of user influence. Proceedings of the Web Technologies and Applications: 14th Asia-Pacific Web Conference, APWeb 2012.

[46] Bakshy E., Hofman J.M., Mason W.A., Watts D.J. Everyone’s an influencer: Quantifying influence on twitter. Proceedings of the Fourth ACM International Conference on Web Search and Data Mining.

[47] Xu W.W., Sang Y., Blasiola S., Park H.W. (2014). Predicting opinion leaders in Twitter activism networks: The case of the Wisconsin recall election. Am. Behav. Sci..

[48] Wang Y., Fikis D.J. (2019). Common core state standards on Twitter: Public sentiment and opinion leaders. Educ. Policy.

[49] Milani E., Weitkamp E., Webb P. (2020). The visual vaccine debate on Twitter: A social network analysis. Media Commun..

[50] Recuero R., Zago G., Soares F. (2019). Using social network analysis and social capital to identify user roles on polarized political conversations on Twitter. Soc. Media Soc..

[51] Haupt M.R., Jinich-Diamant A., Li J., Nali M., Mackey T.K. (2021). Characterizing twitter user topics and communication network dynamics of the “Liberate” movement during COVID-19 using unsupervised machine learning and social network analysis. Online Soc. Netw. Media.

[52] Lamirán-Palomares J.M., Baviera T., Baviera-Puig A. (2019). Identifying Opinion Leaders on Twitter during sporting events: Lessons from a case study. Soc. Sci..

[53] Featherstone J.D., Barnett G.A., Ruiz J.B., Zhuang Y., Millam B.J. (2020). Exploring childhood anti-vaccine and pro-vaccine communities on twitter—A perspective from influential users. Online Soc. Netw. Media.

[54] Casero-Ripollés A. (2021). Influencers in the Political Conversation on Twitter: Identifying Digital Authority with Big Data. Sustainability.

[55] Merrill J.B., Oremus W. (2021). Five Points for Anger, One for a Like: How Facebook’s Formula Fostered Rage and Misinformation.

[56] Liang F., Lu S. (2023). The dynamics of event-based political influencers on Twitter: A longitudinal analysis of influential accounts during Chinese political events. Soc. Media Soc..

[57] Moukarzel S., Rehm M., del Fresno M., Daly A.J. (2020). Diffusing science through social networks: The case of breastfeeding communication on Twitter. PLoS ONE.

[58] Than K., Salamida L. (2023). How Digital Opinion Leaders (DOLs) in Clinical Care are Changing the Medical Landscape. Pharm. Med..

[59] Liao Q.V., Fu W.T. (2014). Age Differences in Credibility Judgments of Online Health Information. ACM Trans. Comput. Hum. Interact..

